# Rationale for the Use of Pirfenidone in Heart Failure With Preserved Ejection Fraction

**DOI:** 10.3389/fcvm.2021.678530

**Published:** 2021-04-22

**Authors:** Francesca Graziani, Rosa Lillo, Filippo Crea

**Affiliations:** ^1^Department of Cardiovascular and Thoracic Sciences, Fondazione Policlinico Universitario A. Gemelli IRCCS, Rome, Italy; ^2^Catholic University of the Sacred Heart, Rome, Italy

**Keywords:** heart failure with preserved ejection fraction, pirfenidone, idiopatic pulmonary fibrosis, inflammation, heart failure

## Abstract

Heart failure with preserved ejection fraction (HFpEF) is a major public health problem with growing prevalence and poor outcomes, mainly due to the lack of an effective treatment. HFpEF pathophysiology is heterogeneous and complex. Recently a “new paradigm” has been proposed, suggesting that cardiovascular and non-cardiovascular coexisting comorbidities lead to a systemic inflammatory state, perturbing the physiology of the endothelium and the perivascular environment and engaging molecular pathways that ultimately converge to myocardial fibrosis. If inflammation and fibrosis are the “*fil rouge*” in the heterogeneous spectrum of HFpEF, anti-fibrotic and anti-inflammatory drugs may have a role in its treatment. Pirfenidone is an orally bioavailable drug with antifibrotic and anti-inflammatory properties already approved for the treatment of idiopathic pulmonary fibrosis. Pirfenidone has been recently tested in animal models of myocardial fibrosis with promising results. Here we will review the rationale underlying the potential therapeutic effect of Pirfenidone in HFpEF.

## Introduction

Heart failure with preserved ejection fraction (HFpEF) is a clinical syndrome characterized by typical symptoms and signs of heart failure (HF) with normal or near-normal left ventricular ejection fraction (LV EF ≥ 50%), echocardiographic features of diastolic dysfunction and/or structural heart disease and elevation of natriuretic peptides (1). The prevalence of HFpEF has grown worldwide and it now represents the dominant form of HF, affecting roughly 5% of the general population aged > 60 years. Contributing factors to this phenomenon are the improvement in diagnostic tools together with the greater clinical awareness and the increase in life expectancy (2). HFpEF patients experience rates of hospitalization, functional decline, and mortality similar to patients with HF and reduced ejection fraction (HFrEF) (3), imposing major economic health care burden. Unlike the established efficacy of several drugs in HFrEF, no specific therapy has yet proven to significantly impact on morbidity and mortality in HFpEF and the current treatment remains “empiric” and mostly symptomatic (4). This unmet need is at least partially explained by the complex and heterogeneous pathophysiology underlying the clinical spectrum of HFpEF. Recently, cardiac fibrosis and microvascular inflammation have emerged as the “*fil rouge*” in the conundrum of HFpEF. Myocardial fibrosis precedes the clinical diagnosis of HFpEF and is strongly associated with disease severity and adverse outcomes (5, 6).

Based on these evidences, a mechanistic overlap between HFpEF and other fibrotic diseases, such as idiopathic pulmonary fibrosis (IPF) seems likely (7). Pirfenidone is an oral anti-fibrotic drug (with also anti-oxidant and anti-inflammatory effects) approved for clinical use in IPF and it can lead to regression of myocardial fibrosis in animal models. We will discuss the rationale underlying the potential therapeutic effect of Pirfenidone in HFpEF.

## Pathophysiology of HFpEF

HFpEF is a systemic syndrome, driven by accumulated risk factors and comorbidities, which, in vulnerable subjects, trigger pathways leading to increased ventricular stiffness, diastolic dysfunction and abnormal ventricular-arterial coupling (8, 9). The underlying mechanisms of diastolic dysfunction are impaired cardiomyocyte relaxation and increased extracellular stiffness, leading to preservation of LV stroke volume at cost of an increase of LV filling pressure (10). Neuro-hormonal system then activates and promotes salt and water retention in the kidney. Over time, the increased circulating volume and high levels of Angiotensin II and aldosterone trigger a maladaptive vicious circle, increasing ventricular stretch, oncostatic pressure in the lungs and peripheries and exerting a pro-hypertrophic and pro-fibrotic effect within the myocardium (11). The original description of HFpEF relied on the relation between arterial hypertension and diastolic dysfunction, with high afterload as trigger of ventricular remodelling and diastolic failure (2). Nowadays it is well-known that HFpEF is a complex disease, and the paradigm of “increased afterload model” is no longer valid.

## Comorbidity-Driven Microvascular Inflammation Theory in HFpEF

**Figure 1:**
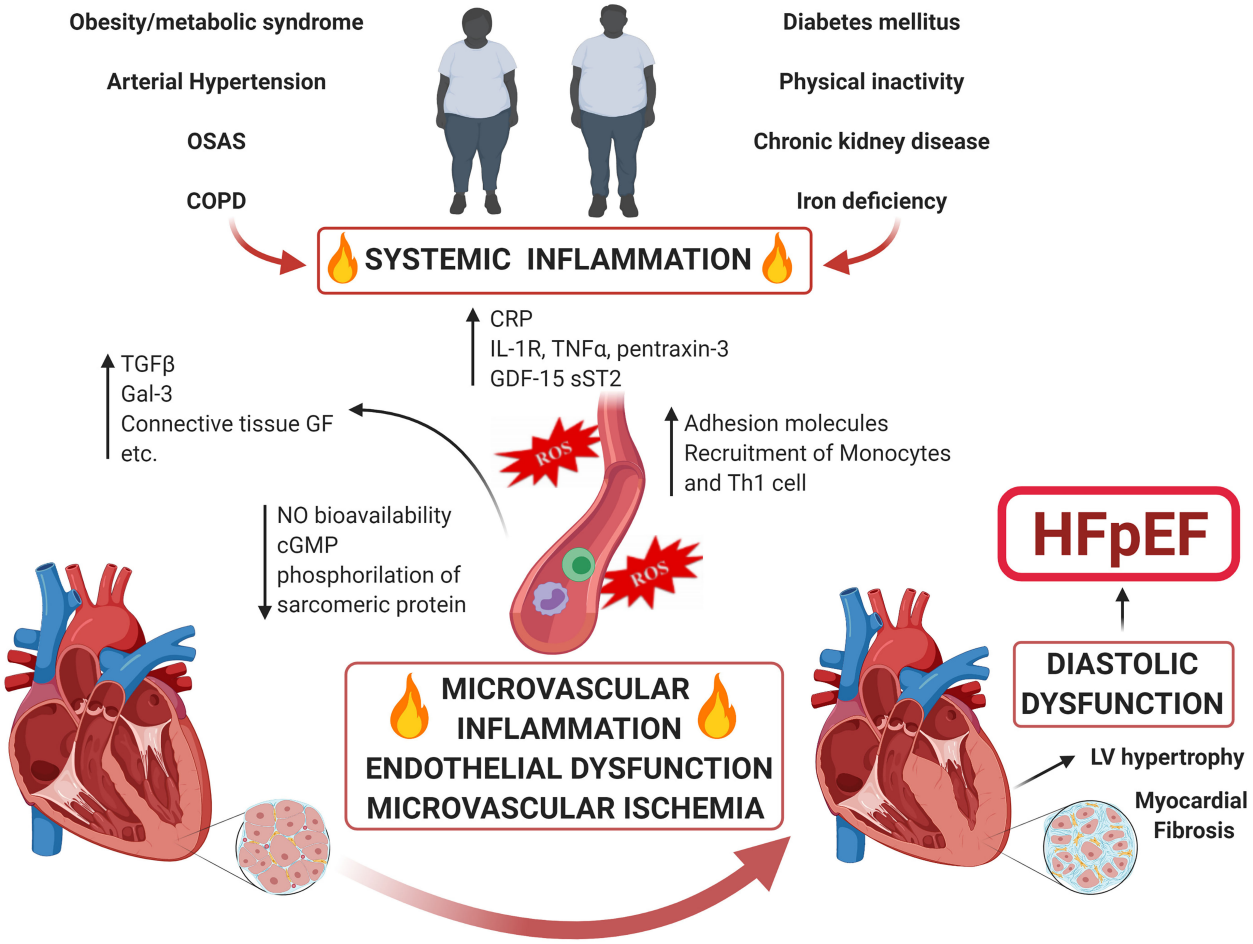
Comorbidity-Driven Microvascular Inflammation Theory in HFpEF. Accumulated risk factors as well as cardiac and non-cardiac comorbidities lead to a systemic inflammatory state and coronary microvascular inflammation. The endothelial dysfunction and perturbation of the physiology of the perivascular environment engage molecular pathways that ultimately converge to microvascular dysfunction and myocardial fibrosis causing HFpEF.

The classic risk factors for developing HFpEF include age, female gender, hypertension, diabetes, overweight/obesity, renal dysfunction, metabolic syndrome (present in the 85% of patients) and physical inactivity (12, 13). According to the “new paradigm” for HFpEF (9) comorbidities induce a systemic pro-inflammatory state, which causes endothelial dysfunction and coronary microvascular dysfunction (CMD) (Figure 1). Under the effect of persistent pro-inflammatory stimuli (suggested by the elevation of circulating inflammatory biomarkers such as IL-1RL1, C-reactive protein, GDF15, TNF-α, sST-2, pentraxin-3, etc.), coronary microvascular endothelium recruits monocytes and Th1 cells through the production of adhesion molecules (14). These inflammatory cells express transforming growth factor β (TGF-β), interferon-ɤ, Galectin-3 (Gal-3), connective tissue growth factor and angiotensin-converting enzymes, promoting the conversion of fibroblasts to myofibroblasts and collagen deposition (9), with TGF-β playing a pivotal role. Moreover, the microvascular endothelial inflammation enhances oxidative stress. This leads to increased reactive oxygen species (ROS) production which perturb nitric oxide (NO) metabolism decreasing its bioavailability, reducing cyclic guanosine monophosphate (cGMP) content and protein kinase G (PKG) activity in adjacent cardiomyocytes, altering the phosphorylation state of sarcomeric proteins and the calcium handling, thus adversely affecting the cardiomyocyte and inducing hypertrophy. This results in increased myocardial stiffness, impaired energetic metabolism and a pro-fibrotic, pro-inflammatory secretome, which contributes to and perpetuates the haemodynamic changes of HFpEF. Histological studies on LV endomyocardial biopsy samples from HFpEF patients showed high level of expression of inflammatory endothelial adhesion molecules including VCAM1, high numbers of CD3, CD11, and CD45-positive leucocytes in the myocardium, increased expression of TGF-β in inflammatory cells and increased levels of collagen I and III (14). Hage et al. (15) found that myeloperoxidase-dependent oxidative stress, reflected by uric acid and calprotectin, is increased in HFpEF patients, suggesting microvascular neutrophil involvement mirroring endothelial dysfunction as a central component of the HFpEF syndrome. Moreover, Pentraxin 3, a biomarker of inflammation, was found to be significantly elevated in HFpEF patients and its levels at the coronary sinus significantly higher than at the aortic root, suggesting a production in the coronary circulation in patients with LV diastolic dysfunction (16). Interestingly, the systemic inflammatory state seems not only to play a pivotal pathophysiological role, but also to have prognostic implications. Levels of Gal-3, a marker of myocardial fibrosis, inversely correlate with functional capacity and its increase over time is associated with a higher risk of death or hospitalization (17). Shah et al. demonstrated that soluble ST2 (member of the IL-1 receptor family) is a strong predictor of mortality in patients presenting with acute dyspnoea and preserved EF (18). Plasma levels of Neopterin, a molecule mainly secreted by activated macrophages, are significantly increased in HFpEF and correlate with the severity of HF and with future cardiovascular events (19).

In acute HFpEF, changes in levels of inflammatory markers such as pentraxin-3, TNF- receptor1a, myeloperoxidase, and lymphotoxin β receptor are correlated to outcome (20). The “new HFpEF paradigm theory” found support also from the results of a proteomic analysis (21): Sanders-van Wijk et al. demonstrated that, across two independent cohorts of HFpEF patients, comorbidity burden was associated with abnormal cardiac structure and function and with increased systemic inflammation, which was associated with worse cardiac function and was upregulated in HFpEF as compared to non-HF controls with comorbidities; importantly, inflammation also appeared to mediate the association between comorbidity burden and worse cardiac haemodynamic.

## Coronary Microvascular Dysfunction and HFpEF

The role of CMD at the basis of HFpEF pathogenesis and evolution has gained growing consent over years (2). CMD is determined by a variable combination of endothelial dysfunction, vascular smooth muscle cell hyper-reactivity, vascular remodelling, fibrosis and rarefaction, and increased extravascular pressure (22). A cross-talk between the endothelium and the surrounding vascular tissue and architecture, as well as the myocardium, seems to play a key role in the pathogenesis of HFpEF. We proposed an innovative theory that identifies CMD as the “common soil” for the occurrence of both microvascular angina (MVA) and HFpEF. Possible modulating factors may determine an effect in one direction or the other. Beyond molecular mediators, an additional potential mechanism, as suggested by Pepine et al. (23) involves recurrent cycles of ischaemia-reperfusion that affect myocyte relaxation leading to diastolic dysfunction and HFpEF (24). In turn, the increased intra-myocardial pressure can enhance myocardial ischemia increasing myocardial oxygen consumption: this can explain the coexistence of a “vicious circle” with subclinical ischaemia directly contributing to the pathogenesis of HFpEF. In support of these considerations, an autoptic analysis of 124 hearts of HFpEF patients demonstrated an inverse relationship between microvascular density and myocardial fibrosis (25). Moreover, CMD is associated with higher left-sided cardiac filling pressures at rest, with this relationship even more pronounced during exercise.

In the multi-national PRevalence Of MIcrovascular dySfunction in Heart Failure with Preserved Ejection Fraction (PROMIS-HFpEF) study (26), CMD has been documented in 75% of HFpEF patients. In the exploratory assessment of the prognosis at 1 year of the PROMIS study population, Hage et al. interestingly found that coexistence of CMD with HFpEF has prognostic implications, being CMD associated with higher incidence rates of CV death/recurrent HF hospitalizations, all-cause death/first HF, and recurrent but not first all-cause hospitalization (27). Taqueti et al. (28), in a study on symptomatic patients without flow-limiting epicardial coronary artery disease, found that impaired coronary flow reserve (CFR) was independently associated with diastolic dysfunction and adverse events, especially HFpEF events: patients with *both* diastolic dysfunction and impaired CFR demonstrated a > 5-fold increased risk of HFpEF hospitalization, providing evidence that CMD, alongside myocardial stiffness, may play an important role in the pathophysiology of HFpEF.

## Myocardial Fibrosis and HFpEF

Myocardial fibrosis is an endogenous response to different cardiac insults that may become maladaptive over time and contribute to the onset and progression of HF. ECM expansion, secondary to excess collagen accumulation, is considered a key pathophysiological mechanism of HFpEF, a common pathway that exists regardless of aetiology. There is strong evidence demonstrating both the primary aetiological role of myocardial fibrosis in HFpEF, but also the adverse impact that ECM expansion has on myocardial mechanical, electrical and microvascular function, confirming that the myocardial fibrotic burden is strongly and independently associated with adverse outcome (29–33). However, there are some conflicting data on the topic, with histological and imaging studies showing that approximately one-third to one-half of HFpEF patients can have normal measures of myocardial fibrosis (34).

Interestingly, myocardial and pulmonary fibrosis share some characteristics and molecular mediators, with TGF-β and Ang II being the major regulating factors. The activation of the AT1 receptor in fibroblasts by Ang II leads to the secretion of TGF-β, which stimulates fibroblast proliferation and ECM synthesis (fibronectin, proteoglycans, and type I–III collagen) in an autocrine manner and induces cardiomyocytes hypertrophy in a paracrine manner (35). TGF-β then activates downstream effectors including smad-dependent and independent signalling pathways. Several studies have also demonstrated the role of other molecular mediators of both cardiac and pulmonary fibrosis, such as sirtuins (Sirt1, Sirt3, Sirt6, Sirt7), MMPs (MMP-9, MMP-13), microRNAs (miRNA 26, miRNA 29) and others (36–47). Interestingly, Cunningham et al. found that circulating biomarkers reflecting mechanisms of ECM homeostasis (sST2), collagen synthesis (PINP, PIIINP), and collagen degradation and turnover (TIMP-1, CITP) are abnormal in patients with HFpEF (48). In such a scenario, it is reasonable to encourage studies aiming to test the potential beneficial effect of anti-fibrotic therapeutic approach in HFpEF patients, learning from the solid experience of treatment for IPF (7).

## Treatment of HFpEF: Knowledge Gaps and Future Perspectives

To date, unlike HFrEF, HFpEF is still orphan of treatments proven to significantly reduce major CV events. Beta-blockers, angiotensin-converting enzyme inhibitors, Ang-II receptor blockers, mineralocorticoid receptor antagonists and angiotensin receptor-neprilysin inhibitors have all failed to reach the pre-specified primary endpoints in trials testing their effects on CV outcomes (Table 1) (49–71), although some have shown improvements in their secondary endpoints.

**Table 1:** Clinical trials of pharmacological therapies for heart failure with preserved ejection fraction.

**Drug**	**Trial**	**Methods**	**Results (primary endpoints)**	**References**
ACEi/ARB	PEP-CHF	Perindopril vs. placebo	No difference in combined all-cause mortality and unplanned HF hospitalization (insufficient power)	(49)
	I-PRESERVE	Irbesartan vs. placebo	No difference in combined death from any cause or hospitalization for a CV cause	(50)
	CHARM-Preserved	Candesartan vs. placebo	Trend towards a reduction in combined CV death or HF hospitalization	(51)
	Enalapril in Older Patients With Heart Failure and Preserved Ejection Fraction	Enalapril vs. placebo	No improvement in exercise capacity or aortic distensibility	(52)
Beta-blockers	ELANDD	Nebivolol vs. placebo	No improvement in 6-min walk test distance	(53)
	J-DHF	Carvedilol vs. placebo	No difference in combined CV death and unplanned HF hospitalization	(54)
MRA	Aldo-DHF	Spironolactone vs. placebo	Improvement in diastolic function (E/e' ratio) but no difference in peak VO2	(55)
	TOPCAT	Spironolactone vs. placebo	No difference in composite outcome of death from CV causes, aborted cardiac arrest, or hospitalization for HF	(56)
ARNI	PARAGON-HF	Sacubitril/Valsartan vs. Valsartan	No difference in combined of CV death and hospitalization for HF	(57)
	PARALLAX	Sacubitril/Valsartan vs. individualized medical therapy	Significant reduction of NTproBNP but no differences in 6-min walk test distance (preliminary results)	(58)
Digoxin	DIG-PEF	Digoxin vs. placebo	No difference in the composite of HF-related hospitalizations and death	(59)
Ivabradine	EDIFY	Ivabradine vs. placebo	No evidence of improvement in any of the three co-primary endpoints: E/e', 6-min walk test distance and NTproBNP reduction	(60)
A1-agonists	PANACHE	Neladenoson bialanate vs. placebo	No significant change in 6-min walk test distance	(61)
Nitrates	NEAT-HFPEF	Isosorbide mononitrate vs. placebo	No increase but rather decrease in daily activity level measured in accelerometer units	(62)
	INDIE-HFPEF	Inhaled nebulized inorganic nitrite vs. placebo	No difference in peak VO2	(63)
PDE-5a inhibitors and sGC activators	RELAX	Sildenafil vs. placebo	No difference in peak VO2	(64)
	Sildenafil on invasive Hemodynamics and exercise capacity in HFpEF and pulmonary Hypertension	Sildenafil vs. placebo	No change in mean pulmonary artery pressure	(65)
	SOCRATES-PRESERVED	Vericiguat vs. placebo	No changes in NTproBNP and left atrial volume	(66)
	VITALITY-HFPEF	Vericiguat vs. placebo	No impovement in physical limitation score of the Kansas City Cardiomyopathy Questionnaire	(67)
	CAPACITY-HFPEF	Praliciguat vs. placebo	No significant improvement in peak VO2	(68)
Anti-inflammatory Drugs	D-HART	Anakinra vs. placebo	Significant improvement in peak VO2 and reduction in plasma CRP levels	(70)
	D-HART 2	Anakinra vs. placebo	No difference in peak VO2 and VE/VCO2 slope	(71)
SGLT2-inhibitors	EMPERIAL-preserved	Empaglifozin vs. Placebo	No difference in 6-min walk test distance (preliminary results)	(72)

Over the last years, the new insights on HFpEF pathophysiology have increased the interest in testing new drugs specifically targeting the molecular mediators involved in HFpEF. Given the role of the oxidative stress and NO metabolism perturbation, it seemed reasonable to propose organic NO donors as potentially useful therapeutic tools. Unfortunately, results from the early studies are at best inconclusive and in some patients, paradoxically, a tendency to reduce the total physical activity was observed (62, 63). However, new trials of oral nitrite and nitrate are currently ongoing. Conflicting data were also reported about Phosphodiesterases-5a (PDE-5a) inhibitors (64, 65). A possible explanation of these unsatisfactory results lies on an inadequate production of endogenous cGMP rather than excessive breakdown by PDE-5 (active synthesis of NO is required). This has led to therapeutic strategies specifically targeting the soluble guanylate cyclase (sGC) using direct sGC stimulators that can increase cGMP production through NO-independent pathways. Vericiguat is a stimulator of sGC tested in the SOCRATES-HFpEF trial (66), the pre-specified primary end-point was the change of NT-proBNP levels or left atrium volume over a 12-week treatment period and the trial failed. Recent data from the VITALITY-HFpEF randomized placebo-controlled trial showed that 24-week treatment with Vericiguat compared with placebo did not improve the physical limitation score (67). Similarly, Praliciguat did not improve significantly the Peak Rate of Oxygen Consumption, thus not supporting its use in patients with HFpEF (CAPACITY HFpEF trial) (68).

Drugs specifically targeting inflammation have been tested in HFpEF: in animal models chemokine antagonists (antiMCP1, MCP3) and immuno-modulatory cytokines (interleukin IL-10, pentraxins, and IL-1b blockade) showed promising results (69). Anakinra, a recombinant IL-1 receptor antagonist, reduced C-reactive protein levels and improved exercise capacity in a crossover trial of 12 patients with HFpEF and elevated C-reactive protein (70). Data from the DHART2 trial, however, showed that Anakinra failed to improve aerobic exercise capacity or ventilatory efficiency in patients with HFpEF, even if high-sensitivity C-reactive protein and NT-proBNP levels were lower after treatment compared with baseline (71).

The sodium–glucose cotransporter 2 (SGLT2) inhibitors (dapaglifozin, empaglifozin, and canaglifozin) were found to reduce hospitalizations as well as mortality in HF (73, 74), but nowadays not conclusive data are available on the effect of these drugs in HFpEF (72) and current evidence does not support a widespread use of these drugs in non-diabetic subjects. In a preclinical study performed in a novel co-culture system combined with a high-throughput analysis of cardiomyocyte function (75) cardiac microvascular endothelial cells exerted a direct positive effect on cardiomyocyte contraction and relaxation, mainly mediated by endothelial-derived NO. This effect is lost after pre-incubation of cardiac microvascular endothelial cells with TNF-α and can be restored with empagliflozin, which leads to restoration of endothelial NO bioavailability. In a study on non-diabetic rat models with HFpEF treated with dapagliflozin this drug ameliorated diastolic function, reversed endothelial activation and endothelial nitric oxide synthase deficit, reducing cardiac inflammation and attenuating pro-fibrotic signalling pathways. The potential involvement of coronary endothelium was supported by the endothelial upregulation of Na+/H+ exchanger 1 *in vivo* and direct effects on dapagliflozin on the activity of this exchanger in endothelial cells demonstrated *in vitro* (76). These data are promising for future investigations.

Due to the key pathophysiological role of myocardial fibrosis in the development and progression of HFpEF, there is a growing interest about the potential beneficial effects of anti-fibrotic drugs, commonly used in other fibrotic disease such as IPF, also in HFpEF such as Pirfenidone.

## Pirfenidone: Pharmacokinetics, Safety Profile, Mechanism of Action

Pirfenidone is an orally bioavailable small synthetic molecule, with proven anti-inflammatory and anti-fibrotic properties and it is authorized by the European Commission for the treatment of adults with IPF (77).

Pirfenidone is rapidly absorbed in the gastrointestinal tract and its half-life is about 3 h (78, 79). It is metabolized in the liver (mainly by CYP1A2) and is mostly excreted as the metabolite 5-carboxy-pirfenidone, by 80% through the urine and by 20% through intestinal elimination. This explains why creatinine clearance <50 mL/min and mild-to-moderate liver dysfunction are relative contraindications to Pirfenidone (78, 79). In the Pirfenidone safety study (PASSPORT) (80) the most frequent side effects documented were nausea and fatigue, gastrointestinal disturbances, skin rash and photosensitivity reactions; serious side effects were rare, with fatal outcome observed in <1%.

**Figure 2:**
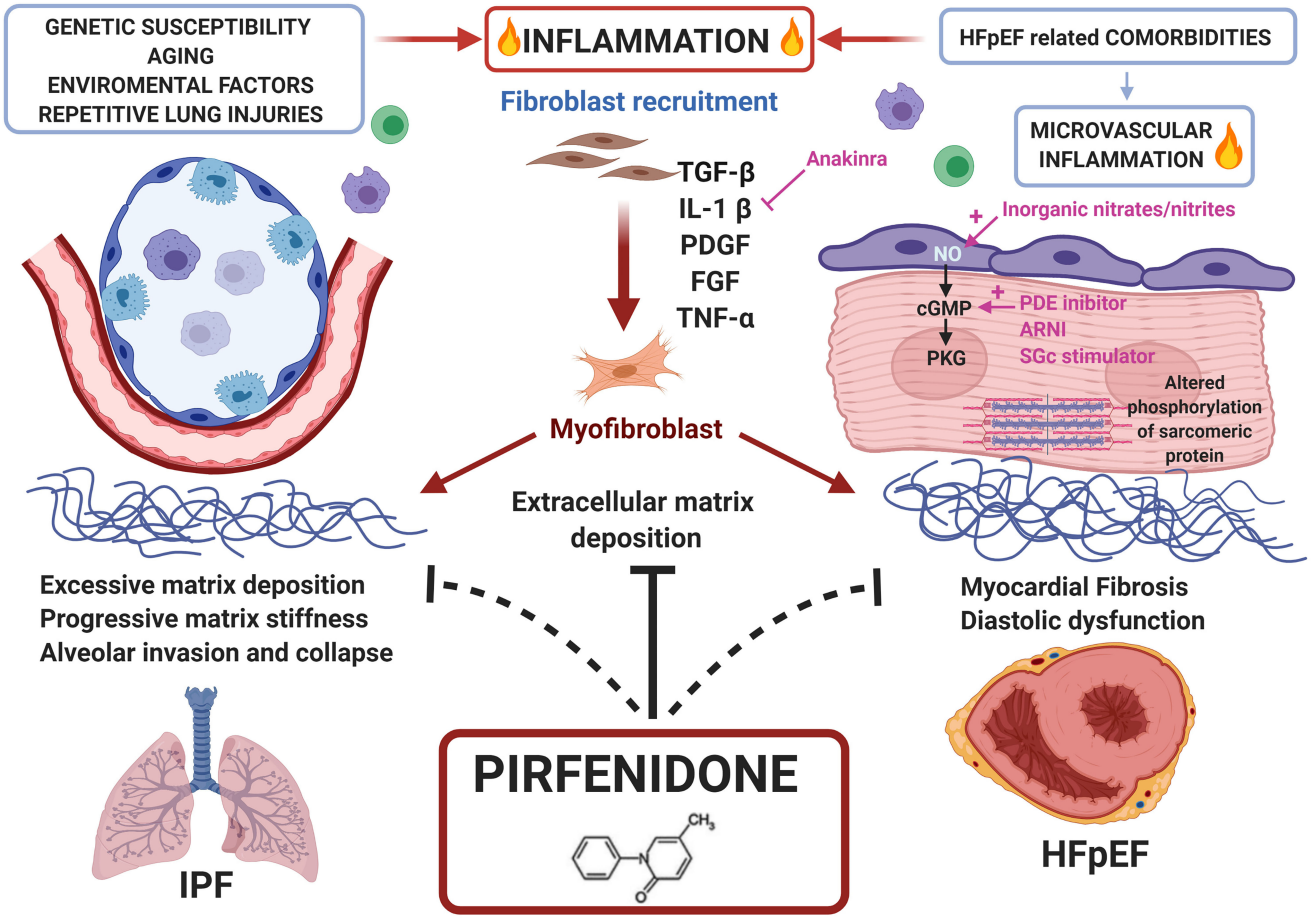
Common pathways between HFpEF and IPF and potential role of Pirfenidone as treatment for both diseases. Repetitive lung injuries over a genetically susceptible alveolar epithelium, activates inflammatory pathways and the overproduction of pro-fibrotic mediators like transforming growth factor (TGF)-β, enhancing fibroblast recruitment, and conversion to myofibroblast. Similarly, HFpEF comorbidities trigger microvascular inflammation converging to myocardial fibrosis. Pirfenidone, an antifibrotic and anti-inflammatory drug approved for clinical use in IPF, may be proposed for HFpEF treatment.

The precise mechanism of action of this drug remains still unclear (77). Pirfenidone attenuates fibroblast proliferation, production of fibrosis-associated proteins (TGF-β, platelet-derived growth factor and β fibroblast growth factor) and cytokines (interleukin-1β and tumour necrosis factor-α), and reduces the increased biosynthesis and accumulation of extracellular matrix in response to pro-fibrotic mediators (i.e., TGF-β); it also blocks the proliferation and differentiation of fibroblasts into myofibroblasts by inhibiting several targets of TGF-β (Smad3, p38, Akt42), improves mitochondrial function and modulates lymphocyte activation (79). Pirfenidone has proven clinical effectiveness in IPF. Given the molecular overlap between pro-fibrotic pathways in lung and heart disease and the pleiotropic effects of Pirfenidone, this drug is being considered with increasingly interest as a potential treatment for cardiac disorders (7, 79) (Figure 2).

## Pirfenidone and Myocardial Fibrosis: What Do We Know?

The available data on cardio-protective effects of Pirfenidone are still in a preclinical phase, but there are several evidences pointing to that direction (79).

Pirfenidone, in a dose- and time-dependent manner, reduces *cardiac* fibroblasts migratory ability, inhibits their proliferation and the process of myofibroblast differentiation (by inhibition of α-SMA expression) as well as the myocardial fibroblast synthesis and secretion of TGF-β1. Pirfenidone also regularizes ratios of myocardial MMPs and tissue inhibitors of metalloproteinases, enhancing myocardial renin-angiotensin system imbalance and cardiac fibroblast synthesis and secretion of IL-10, an anti-fibrotic cytokine (81–88).

In hypertensive mouse models (87), Pirfenidone showed to reverse and prevent cardiac remodelling and the increased cardiac stiffness. Similarly, Yamazaki et al. (88) found that Pirfenidone can prevent the progression of Ang II-induced cardiac hypertrophy and fibrosis, and Yamagami et al. (89) demonstrated that myocardial inflammation was alleviated in mice exposed to transverse aortic constriction. The Pirfenidone power to reduce cardiac fibrosis has been documented in streptozotocin-induced diabetes mice (86) and rats receiving intraperitoneal injections of doxorubicin too (83). In dog models (90) with HF induced by high-frequency left ventricular pacing, Pirfenidone showed a protective effect, preventing fibrosis of the atrial myocardial tissue. In a rat model (91) of myocardial infarction, Pirfenidone decreased scar size and myocardial fibrosis in the border zone, improving left ventricular systolic function, and reduced ventricular tachycardia susceptibility, suggesting a potential role of this drug also in this setting.

## Pirfenidone and HFpEF: A Current Gap of Knowledge

To date, no data on Pifenidone effect on human myocardial fibrosis are available. Despite the prominent role of fibrosis in the pathophysiology of several cardiac disorders, and the evidence of the safety of therapy with Pifenidone, only a single study on the use of Pirfenidone for a cardiac condition has been started, the Efficacy and Safety of Pirfenidone in Patients With Heart Failure and Preserved Left Ventricular Ejection Fraction (PIROUETTE) trial (92). This is a randomised, double-blind, placebo-controlled phase II trial evaluating the efficacy and safety of 52 weeks of treatment with Pirfenidone in patients with HFpEF and myocardial fibrosis (defined as extracellular matrix volume ≥ 27% measured with cardiovascular magnetic resonance). The primary outcome of the study is the change in myocardial ECM volume. A sub-study will also investigate the relationship between myocardial fibrosis and myocardial energetics, and the related impact of Pifenidone. The trial is still ongoing and there is a growing interest about its results, it could lay the foundation for the improvement of outcome in HFpEF patients.

In humans, only two retrospective studies have investigated the effect of Pirfenidone on LV structure and function in patients with IPF treated with this drug.

In the first one, Alansari et al. (93) hypothesized that Pirfenidone could have had a more favourable effect on changes in echocardiographic parameters of LV structure and function in IPF patients responder compared to non-responders (defined as an absolute decline in forced vital capacity of more than 10% whilst being on the medication). After treatment, no significant differences in changes of echocardiographic parameters of LV structure, diastolic function, systolic function and GLS were observed between the two groups. In the second one (94), the same authors found that treatment with Pirfenidone was associated with decreases in indexed LV end diastolic and end systolic volumes. However, no improvements were noted in markers of LV diastolic, systolic function and strain. Both studies, however, were retrospective and meaningfully limited by their small sample size (27 and 24 subjects, respectively), while large and specifically designed prospective trials are needed to test properly the efficacy of Pirfenidone in HFpEF.

If cardiac fibrosis and microvascular inflammation are the common pathophysiological substrates in this conundrum, Pirfenidone could have a powerful role in the treatment of all the different HFpEF subtypes, targeting the “core mechanisms” they all share. However, differences in timing of fibrogenesis and in fibrotic burden in each HFpEF-related comorbidity are still poorly understood, being influenced by many co-factors. Nowadays circulating biomarkers are considered a powerful tool to depict the patients pro-inflammatory and pro-fibrotic profile. Moreover, CMR offers the unique possibility to non-invasively estimate the amount of myocardial fibrosis. We can speculate that a “risk stratification” strategy, using inflammation and fibrogenesis biomarkers and CMR, might identify patients who will benefit most from Pirfenidone: those showing an intense inflammatory and fibrogenetic activation before the evolution toward an advanced, irreversible, stage of diffuse myocardial fibrosis. Trials specifically testing Pirfenidone effects on patients with this profile might help to optimize treatment in HFpEF.

## Conclusions

Up to now, HFpEF treatment has been borrowed from the HFrEF experience, with poor results. The complex and not completely understood HFpEF pathophysiology is probably the key to develop a tailored effective treatment. The new HFpEF paradigm states that the coronary microvascular endothelial inflammation is the main driving factor, activating complex molecular pathways that eventually converge to myocardial fibrosis. Coronary microvascular inflammation and myocardial fibrosis can be considered the *fil rouge* in the HFpEF conundrum, thus they can be considered reasonable targets treatment. Pirfenidone is a well-established drug for the treatment of IPF and in animal studies it showed its anti-inflammatory properties and its ability to reverse cardiac fibrosis. Taken together these data suggest that Pirfenidone could have a role in the treatment of HFpEF by targeting inflammation and myocardial fibrosis, however, at present, clinical trials are lacking. Large, specifically-designed studies with hard end-point in this setting are needed.

## Author Contributions

FG and RL wrote sections of the manuscript. FC contributed to manuscript revision. All authors read and approved the submitted version.

## Conflict of Interest

The authors declare that the research was conducted in the absence of any commercial or financial relationships that could be construed as a potential conflict of interest.
